# Systemic dendrimer-drug nanomedicines for long-term treatment of mild-moderate cerebral palsy in a rabbit model

**DOI:** 10.1186/s12974-020-01984-1

**Published:** 2020-10-25

**Authors:** Zhi Zhang, Yi-An Lin, Soo-Young Kim, Lilly Su, Jinhuan Liu, Rangaramanujam M. Kannan, Sujatha Kannan

**Affiliations:** 1 Department of Anesthesiology and Critical Care, Johns Hopkins School of Medicine, Baltimore, MD USA; 2 Present address: Department of Natural Sciences, University of Michigan-Dearborn, Dearborn, MI USA; 3 Center for Nanomedicine, Wilmer Eye Institute, Johns Hopkins School of Medicine, 400 North Broadway, Baltimore, MD 21287 USA; 4 Department of Anesthesiology and Critical Care Medicine, Charlotte Bloomberg Children’s Center 6318D, 1800 Orleans Street, Baltimore, MD 21287 USA

**Keywords:** Cerebral palsy, Neuroinflammation, Microglia, Neurobehavior, PAMAM dendrimers, NAC

## Abstract

**Background:**

Neuroinflammation mediated by microglia plays a central role in the pathogenesis of perinatal/neonatal brain injury, including cerebral palsy (CP). Therapeutics mitigating neuroinflammation potentially provide an effective strategy to slow the disease progression and rescue normal brain development. Building on our prior results which showed that a generation-4 hydroxyl poly(amidoamine) (PAMAM) dendrimer could deliver drugs specifically to activated glia from systemic circulation, we evaluated the sustained efficacy of a generation-6 (G6) hydroxyl-terminated PAMAM dendrimer that showed a longer blood circulation time and increased brain accumulation. *N*-acetyl-l-cysteine (NAC), an antioxidant and anti-inflammatory agent that has high plasma protein binding properties and poor brain penetration, was conjugated to G6-PAMAM dendrimer-NAC (G6D-NAC). The efficacy of microglia-targeted G6D-NAC conjugate was evaluated in a clinically relevant rabbit model of CP, with a mild/moderate CP phenotype to provide a longer survival of untreated CP kits, enabling the assessment of sustained efficacy over 15 days of life.

**Methods:**

G6D-NAC was conjugated and characterized. Cytotoxicity and anti-inflammatory assays were performed in BV-2 microglial cells. The efficacy of G6D-NAC was evaluated in a rabbit model of CP. CP kits were randomly divided into 5 groups on postnatal day 1 (PND1) and received an intravenous injection of a single dose of PBS, or G6D-NAC (2 or 5 mg/kg), or NAC (2 or 5 mg/kg). Neurobehavioral tests, microglia morphology, and neuroinflammation were evaluated at postnatal day 5 (PND5) and day 15 (PND15).

**Results:**

A single dose of systemic ‘long circulating’ G6D-NAC showed a significant penetration across the impaired blood-brain-barrier (BBB), delivered NAC specifically to activated microglia, and significantly reduced microglia-mediated neuroinflammation in both the cortex and cerebellum white matter areas. Moreover, G6D-NAC treatment significantly improved neonatal rabbit survival rate and rescued motor function to nearly healthy control levels at least up to 15 days after birth (PND15), while CP kits treated with free NAC died before PND9.

**Conclusions:**

Targeted delivery of therapeutics to activated microglia in neonatal brain injury can ameliorate pro-inflammatory microglial responses to injury, promote survival rate, and improve neurological outcomes that can be sustained for a long period. Appropriate manipulation of activated microglia enabled by G6D-NAC can impact the injury significantly beyond inflammation.

## Background

Neuroinflammation plays an important role during the progression of many disorders in the central nervous system (CNS), including cerebral palsy (CP) [1, 2]. Following brain injury, microglia serves as a crucial pathological mediator that regulates the inflammation process and tissue remodeling [3–5]. However, excessive pro-inflammatory activation of microglia can lead to the over-production of free radicals, excitotoxic metabolites, and pro-inflammatory cytokines, subsequently amplifying inflammatory responses and resulting in impaired repair and worsening brain injury [6–10]. Maternal infection/immune activation and fetal or neonatal inflammation have been implicated in the development of neurologic disorders such as CP, autism, and learning disabilities [11–14]. We have previously demonstrated that intrauterine inflammation in pregnant New Zealand White rabbits results in hind limb spasticity and motor deficits, glutamate excitotoxicity, periventricular white matter injury, and impaired development of thalamocortical fibers and somatosensory cortex, as seen in patients with cerebral palsy, that is mediated by a prolonged and robust pro-inflammatory microglial activation in the periventricular region of the brain in the neonatal rabbits [15–21]. Therefore, mitigation of microglia-mediated inflammation can provide an effective strategy to slow the disease progression and improve neurological outcomes.

Delivering drugs to specifically manipulate the immune response has been a challenge since many of the agents do not cross the BBB. Drugs that do cross the BBB typically do not accumulate in the target cells, often leading to off-target effects in the brain and other organs [22]. NAC, a promising clinically approved anti-inflammatory and anti-oxidant drug, exhibits high plasma protein binding during systemic circulation, relatively poor brain penetration, and lacks CNS targeting properties. We and others have shown that NAC, and its de-acetylated active moiety l-cysteine, is transported through the cystine-glutamate antiporter (System Xc-) that is upregulated in the presence of inflammation and can lead to increased extracellular glutamate potentially worsening excitotoxic injury [23, 24]. Dendrimers bypass standard drug uptake and transport mechanisms providing significant avenues for targeting pathological cells in the CNS while delivering a large drug payload to the target cell [25–29]. Hydroxyl-terminated polyamidoamine (PAMAM) dendrimers are non-toxic, non-immunogenic, and biocompatible [30]. The surface of hydroxyl-terminated PAMAM dendrimers is nearly neutral, which avoids strong interactions with serum proteins during systemic circulation [31]. These PAMAM dendrimers are small enough (~ 4–6 nm) to penetrate the disrupted BBB in the presence of neuroinflammation and diffuse well in the brain parenchyma enabled by the hydroxyl groups [32]. We have previously described the use of generation 4 hydroxyl-terminated PAMAM dendrimer-NAC conjugate (G4D-NAC) to deliver therapeutics specifically into activated microglia near the injured regions for in vivo models of CNS disorders, including white matter injury [33], hypothermic cardiac arrest induced brain injury [23, 34], glioblastoma [35], Rett syndrome [24], and CP [31, 32].

In this study, we explored the efficacy following a single dose of NAC, delivered by a higher generation 6 hydroxyl-terminated PAMAM dendrimer (G6-OH). G6-OH has shown prolonged systemic circulation and enhanced brain accumulation because their size (6.7 nm) lies in the range that avoids rapid glomerular filtration for renal clearance (< 6 nm) [36]. We have previously shown that G6-OH has a longer serum circulation time than G4-OH and greater accumulation in the brain [36, 37]. Using a rabbit model of maternal inflammation-induced mild/moderate phenotype of cerebral palsy, we demonstrate in this study that intravenous treatment on PND1 with a single dose of G6-OH dendrimer NAC conjugate, containing a very low dose of NAC, decreased microglial activation and pro-inflammatory cytokine levels on PND5 and improved survival and motor function to healthy control level up to at least 15 days (PND15). These effects are achieved due to the longer circulation time of the G6-OH dendrimer, resulting in increased NAC accumulation in the activated microglia.

## Materials and methods

### Synthesis and purification

#### 2-(pyridyl-disulfanyl)ethanol

All reagents were purchased from Sigma-Aldrich unless noted. To a solution of Aldrithiol-2 (1.213 g, 5.506 mmol) that was dissolved in MeOH (3 mL), 2-mercaptoethanol (212 μL, 2.25 mmol) was added dropwise, and the reaction mixture was stirred at room temperature overnight. The solvent was then removed by a rotary evaporator under reduced pressure to yield the crude product as yellowish oil. The residue was then purified by flash column chromatography—eluted with hexane/EtOAc 5:3—on a CombiFlash® Rf+ purification system (Teledyne Isco, Lincoln, NE) to yield 2-(pyridyl-disulfanyl)ethanol as pale yellowish oil (438 mg, 78.5 %). ^1^H NMR (DMSO-G6D-NAC, 500 MHz): δ_H_ (ppm) = 2.93 (t, 2H, J = 6.29 Hz), 3.61–3.65 (m, 2H), 5.00 (t, 1H, J = 5.66 Hz), 7.23–7.33 (m, 1H), 7.75–7.90 (m, 2H), 8.45–8.55 (m, 1H); ^13^C NMR (DMSO-G6D-NAC, 125 MHz) δ_C_ (ppm) = 41.2, 59.1, 119.3, 121.1, 137.8, 149.5, 159.5.

#### 4-nitrophenyl(2-pyridin-2-yldisulfanyl)ethyl carbonate

The synthesis of this compound was based on a modified protocol of a reported work [38]. To a solution of 4-nitrophenylchloroformate (3.208 g, 15.87 mmol) that was dissolved in anhydrous DCM (15 mL), 2-(pyridyldisulfanyl)ethanol (1.981 g, 10.58 mmol) and pyridine (1.3 mL, 15.87 mmol) that were dissolved in anhydrous DCM (10 mL) were added dropwise under N_2_ gas at room temperature which yielded a cloudy solution. After 6 h, additional 4-nitrophenylchloroformate (1.07 g, 5.29 mmol) and pyridine (0.43 mL, 5.29 mmol) were dissolved in anhydrous DCM (10 mL) and added to the reaction mixture. The reaction continued at room temperature overnight. The obtained solution was washed with 1 M HCl (60 mL) three times. The organic layer was then collected, dried over Na_2_SO4, filtered, and then concentrated under reduced pressure. The obtained residue was purified with flash column chromatography—eluted with a gradient of hexane/EtOAc from 20:3 to 10:3—on a CombiFlash® Rf+ purification system (Teledyne Isco, Lincoln, DE) to yield 4-nitrophenyl(2-pyridin-2-yldisulfanyl)ethyl carbonate as pale yellowish oil (3.195 g, 9.060 mmol, 85.6%). ^1^H NMR (CDCl_3_, 500 Hz, Me_4_Si): δ_H_ (ppm) = 3.17 (t, J = 6.45 Hz, 2H), 4.57 (t, J = 6.45 Hz, 2H), 7.12–7.16 (m, 1H), 7.35–7.41 (m, 2H), 7.64–7.70 (m, 2H). 8.26–8.31 (m, 2H), 8.49–8.52 (m, 1H); ^13^C NMR (CDCl_3_, 125 MHz, Me_4_Si): δ_C_ (ppm) = 6.8, 66.7, 120.3, 121.2, 121.8, 125.4, 137.2, 149.8, 152.2, 155.4, 159.1.

#### G6D-NAC

4-nitrophenyl(2-pyridin-2-yldisulfanyl)ethyl carbonate (702 mg, 2.00 mmol), 4-dimethylaminopyridine (243 g, 1.99 mmol), and generation 6 hydroxy-terminated PAMAM dendrimers (G6D-OH, 450 mg, 0.00772 mmol; Dendritech, Inc., Midland, MI) that were dissolved in anhydrous DMF (20 mL) were added to an oven-dried flask under N_2_ gas at 40 °C. The reaction continued at 40 °C for 48 h. The solution was then dialyzed against DMF (MWCO 8kD) for 24 h for the removal of excess reagents and side products, which gave a yellowish solution containing the intermediate G6D-PDP that was subsequently transferred to another round bottom flask for the following reaction. To this solution, *N*-acetyl-l-cysteine (200 mg, 1.23 mmol) that was dissolved in anhydrous DMF (4 mL) was added dropwise under N_2_ gas. The reaction was carried out at room temperature overnight. The solution was then dialyzed against DMF (MWCO: 8 kD) for 24 h to give a colorless solution, and then it was titrated in chilled ethyl ether (100 mL) to form a cloudy suspension. The precipitates were collected, subsequently washed with ethyl ether (50 mL) at least 2 times, and then dried under reduced pressure overnight. The dried solids were dissolved in DI H_2_O (30 mL), dialyzed against DI H_2_O for 4 h (MWCO, 8 kD), and then lyophilized to yield the dry mass of G6D-NAC as an off-white solid (556 mg). The purity of the dendrimer conjugates were assessed by analytical RP-HPLC (Waters Corporation, Milford, MA) using 0.1% TFA H2O/ACN as eluting solvents. ^1^H NMR (DMSO-G6D-NAC, 500 Hz): δ_H_ (ppm) = 1.87 (s, CH_3_ of NAC), 2.26 (bs, CH_2_ of G6-OH), 2.60–2.88 (m, CH_2_ of G6-OH), 2.88–3.46 (m, CH_2_ of G6-OH and linker), 4.19 (d, CH_2_OC=OOCH_2_, linker), 4.40 (s, CH of NAC), 7.7–8.4 (m, NHC=O protons of G6-OH).

### Release studies

The release of NAC-associated derivatives was monitored by reverse phase HPLC (Waters Corporation, Milford, MA) using 0.1% TFA H_2_O/ACN as eluting solvents. A solution of G6D-NAC was mixed with glutathione (GSH) (Sigma-Aldrich, St. Louis, MO) to make up a final solution (in DPBS) of 3 mg/mL G6D-NAC with 10 mM GSH. As a comparison, another G6D-NAC solution was prepared at 3 mg/mL in DPBS without the addition of GSH to serve as the control. Both solutions were placed on a horizontal shaker and incubated at 37 °C. For both solutions, 60 μL of solutions were taken out after 0 h (immediately after mixing), 1, 2, 4, 6, 8, 24, and 48 h and then immediately flash frozen. Solutions were kept at – 80 °C until they were analyzed by RP-HPLC. The amount of each NAC-associated species was quantified by measuring the area under the curve in the chromatograms monitored by a photodiode array (PDA) detector at 220 nm.

### Dynamic light scattering and zeta potential

The hydrodynamic diameter and zeta potential of G6D-NAC were measured on a Zetasizer Nano ZS (Malvern Instrument Inc., Westborough, MA). A solution of G6D-NAC in DPBS (0.5 mg/mL) was filtered with a 0.2 μm Acrodisc® syringe filter (Pall Corporation, Port Washington, NY) prior to being loaded in the cuvettes for measurement. Solutions were allowed to stabilize at 25 °C for 2 min prior to the measurements.

### Cytotoxicity assay

Murine brain microglial cells (BV-2) from ATCC were cultured in Dulbecco’s modified Eagle’s medium (DMEM supplemented with FBS and penicillin/streptomycin). BV-2 cells were seeded at a concentration of 10,000 cells/well in a 96-well plate and incubated at 37 °C for 24 h. The cells were treated with 20 ng of LPS and incubated for 3 , which induced activation of microglial cells. Subsequently, the cells were treated with medium containing different concentrations of G6D-NAC or NAC and incubated for additional 24 h. Cells only treated with media served as the control. The cell viability was assessed by using MTS cell proliferation assay kit. The absorbance was detected at 540 nm by a microplate reader. The cell viability was presented as the percentage relative to the absorbance measured from the control cells.

### In vitro anti-inflammatory assay

BV-2 cells were seeded at a concentration of 10,000 cells/well in a 96-well plate and incubated at 37 °C for 24 h in the Dulbecco’s modified Eagle’s medium (DMEM supplemented with FBS and penicillin/streptomycin). The cells were pretreated with 100 ng/mL of LPS and resulted into the amoeboid morphology. After 3 h of incubation, NAC or G6D-NAC solution was added into the wells at the desired concentration. After additional 3 h, the media containing LPS and NAC/G6D-NAC were removed. Each well was rinsed with PBS, fresh media was added, and the cells were incubated for additional 24 h. Nitrite levels were determined by an ELISA kit (Promega, Madison, WI) based on the calibration curve of absorbance at 450 nm.

### Rabbit model of CP and administration of dendrimers

Timed-pregnant New Zealand white rabbits were purchased from Robinson Services Inc. (North Carolina, USA) and arrived at the facility 1 week before surgery. All animals were housed under ambient conditions (22 °C, 50% relative humidity, and a 12-h light/dark cycle), and necessary precautions were undertaken throughout the study to minimize pain and stress associated with the experimental treatments. Experimental procedures were approved by the Johns Hopkins University Animal Care and Use Committee (IACUC). After 1 week of acclimation, the pregnant rabbits underwent laparotomy on gestational day 28 (G28) and received a total of 3200 EU of LPS (lipopolysaccharide; *E. coli* serotype O127:B8, Sigma Aldrich, St Louis MO) injection along the wall of the uterus as previously described [20, 39]. The control group did not receive any intervention. The kits from both groups were induced on G30 with intravenous injection of Pitocin (0.2 unit/kg) and kept in incubators with the temperature of ~ 32–35 °C and a relative humidity of ~ 50–60%.

The littermates from the CP group were randomly divided into 5 groups: PBS, NAC2, NAC5, G6D-NAC2, and G6D-NAC5. On postnatal day (PND1), kits received systemic administration (i.v.) of PBS, 2.0 mg/kg NAC, 5.0 mg/kg NAC, 2.0 mg/kg G6D-NAC, or 5.0 mg/kg G6D-NAC, respectively. The kits from the control group did not receive any intervention and served as healthy controls. All solutions used for administration were sterilized using 0.2 μm Acrodisc® syringe filters (Pall Corporation, Port Washington, NY) prior to injection.

### Behavioral testing

The animals’ general physical conditions (e.g., weight gain, food intake) were monitored daily. Neurobehavioral tests were carried out on PND1 before drug administration (baseline, 0 h), as well as 24, 48, and 96 h post-drug administration by personnel blinded to the experiments. Each animal was videotaped for 10 min and scored on a scale of 0–3 (0 = worst; 3 = best) for movements of head and limbs on a flat surface as previously described for rabbits [16, 40, 41]. The kits were fed with Wombaroo rabbit milk replacer (Perfect Pets Inc., Belleville, MI), and the suck/swallow and head turn during feeding were assessed on a scale of 0–3 (worst–best) [16, 40, 41]. The muscle tone of each limb was scaled at 0–4 (0 = no increase in tone; 4 = limb rigid in flexion or extension) [40]. To minimize the impacts of disease phenotype variability, the changes in the behavioral scores before (0 h) and 24, 48, and 96 h post-treatment for each kit were used to evaluate the efficacy of therapies. In details, the behavioral scores of each kit on PND1 before treatment (0 h) were used as the baseline scores. The changes in the neurobehavioral scores at 24, 48, and 96 h post-treatment for each kit were calculated as:

$$ {\displaystyle \begin{array}{l} Changes\ {from\ baseline}_{(48)}={Score}_{\left( 48\ h\right)}\hbox{-} {Score}_{\left( 0\ h\right)} or\ Changes\ {from\ baseline}_{(96)}={Score}_{(96h)}\hbox{-} \\ {}{Score}_{\left( 0\ h\right)}\end{array}} $$

The changes of all the kits in each group were averaged and compared among groups.

### Locomotor activity

The locomotor activity of the rabbit kits was assessed with EthoVision XT (Noldus Information Technology Inc., Leesburg, VA, USA). The open arena was 65 × 65 × 35 cm. On PND15, rabbit kits were transferred to the apparatus and the spontaneous locomotor activity was recorded for 10 min. The recording was performed at 10 AM, and the animals were not disturbed during the recording. Following the recording, the rabbit kits were returned to their cages. The distance traveled (cm), velocity (cm/s), immobile time (s), and time spent in the center of the arena (s) were measured and compared between control and endotoxin groups.

### Immunohistochemistry

To evaluate the microglia morphology at P5 and P15, rabbits were anesthetized and transcardially perfused with PBS, followed by 10% formalin. The brains were removed and post-fixed in 10% formalin overnight and cryoprotected in graded sucrose solutions. Coronal sections (30 μm, 1:6 series) were incubated in 0.3% hydrogen peroxide (H_2_O_2_) solution, blocked by 3% normal donkey serum in 0.1 M phosphate-buffered saline (PBS). Sections were then incubated overnight at 4 °C with goat anti-IBA1 (ionized calcium binding adaptor molecule 1; 1:500, Abcam, MA. USA). Sections were subsequently washed and incubated with biotinylated secondary antibodies (1:250; Vector Laboratories, Burlingame, CA) for 4 h at room temperature. Next, the sections were incubated for 2 h in an avidin–biotin substrate (ABC kit, Vector Laboratories, Burlingame, CA). All sections were then incubated for 2 min in 3,30-diaminobenzidine (DAB) solution (Vector Laboratories). Sections were dehydrated in ethanol and Histo-Clear^TM^ II (Fisher Scientific, Pittsburg, PA, USA) and cover-slipped using mounting medium. To evaluate the co-localization of G6-DNAC-Cy5, TSPO (translocator protein), and microglia in healthy control and CP kits, the brain sections were incubated overnight at 4 °C with goat anti-TSPO (1:250, Abcam, MA, USA). Sections were subsequently washed and incubated with DyLight 594-labeled lycopersicon esculentum lectin (Victor laboratories, Inc., CA, USA) and fluorescent secondary antibodies (1:250; Life Technologies, MA, USA) for 2 h at room temperature. Next, the sections were incubated with DAPI (1:1000, Invitrogen) for 15 min. After washing, the slides were dried and cover-slipped with mounting medium (Dako, Carpinteria, CA, USA). Confocal images were acquired with a Zeiss ZEN LSM 710 (Zeiss, CA, USA) and processed with ZEN software.

### Microglial morphological analysis

All slides and images were coded, and the analysis was performed with the personnel blinded to experiments. Images (× 40, 4–6 images/animal) were randomly acquired from the corpus callosum and cerebellar white matter areas using Nikon Eclipse 90i and Stereo Investigator software (MBF Bioscience, Williston, VT, USA). Microglia to be traced (× 40, 1–2 cells/image) were chosen at random from the corpus callosum and cerebellar white matter areas. The microglia that met the following criteria were traced: (1) cell body located in the corpus callosum; (2) processes completely contained within the slice; and (3) cells sufficiently stained to allow for tracing processes. The soma morphology and processes structures of the microglia were analyzed using the Neurolucida Explorer software package (MBF Bioscience, Williston, VT, USA).

### Real-time PCR

The brains were quickly harvested and stored in RNAlater solution (Life technologies, Grand Island, NY, USA). The periventricular region (PVR) white matter and cerebellar white matter areas were micro-dissected (~ 50 mg). The total RNA was extracted using TRIZOL (Life Technologies, Grand Island, NY, USA) according to the manufacturer’s instructions. RNA samples were quantified using the Nanodrop ND-1000 Spectrophotometer (Thermo Fisher Scientific, Walkersville, MD). The single-stranded complementary DNA (cDNA) was first reverse-transcribed from the total RNA samples using the High-Capacity cDNA Reverse Transcription Kit with RNase inhibitor (Life Technologies, Grand Island, NY, USA). The real-time PCR was performed with Power SYBR® Green PCR Master Mix (Life Technology, Grand Island, NY, USA) using Fast 7500 Real-time PCR systems (Life Technologies, Grand Island, NY, USA). Amplification conditions included 30 min at 48 °C, 10 min at 95 °C, 40 cycles at 95 °C for 15 s, and 60 °C for 1 min. Primers were custom-designed (Table 1) and ordered from Integrated DNA Technology (Iowa, USA). Comparative Ct method was used to assess differential gene expressions. The gene expression levels for each sample were normalized to the expression level of the housekeeping gene encoding glyceraldehyde 3-phosphate dehydrogenase (GAPDH) within a given sample (ΔCt); the differences between the treatment groups to the healthy control group were used to determine the ΔΔCt. The 2^−ΔΔCt^ gave the relative fold changes in gene expression.

**Table 1:** Primers for real-time-PCR

Gene	Forward primer	Reverse primer
TNF-α	TAGTAGCAAACCCGCAAGTG	CTGAAGAGAACCTGGGAGTAGA
IL-1β	TGCCAACCCTACAACAAGAG	AAAGTTCTCAGGCCGTCATC
IL-10	CCTGTGGGATTTGAGTGTCTTA	GCTCGGCTTAGGAGTTAGAAAG
TGF-β1	TGAGAGGTGGAGAGGAAATAGA	GGAACTGATCCCGTTGATGT
TSPO	ACT TGA ACC TTC ACC CAT GCA GGA	AAG CAG TTA CAG AGG GAG CGT
GAPDH	TGA CGA CAT CAA GAA GGT GGT G	GAA GGT GGA GGA GTG GGT GTC

### Statistical analysis

All data were presented as mean ± SEM. Statistical analysis was conducted by GraphPad Prism (version 6.07). Two-tailed Student’s *t* test was used for two group comparisons, and one-way ANOVA with Fisher’s post hoc analysis was used for multi-variant comparisons. A value of *p* < 0.05 was considered statistically significant.

## Results

### Synthesis and characterization of G6D-NAC

**Fig. 1:**
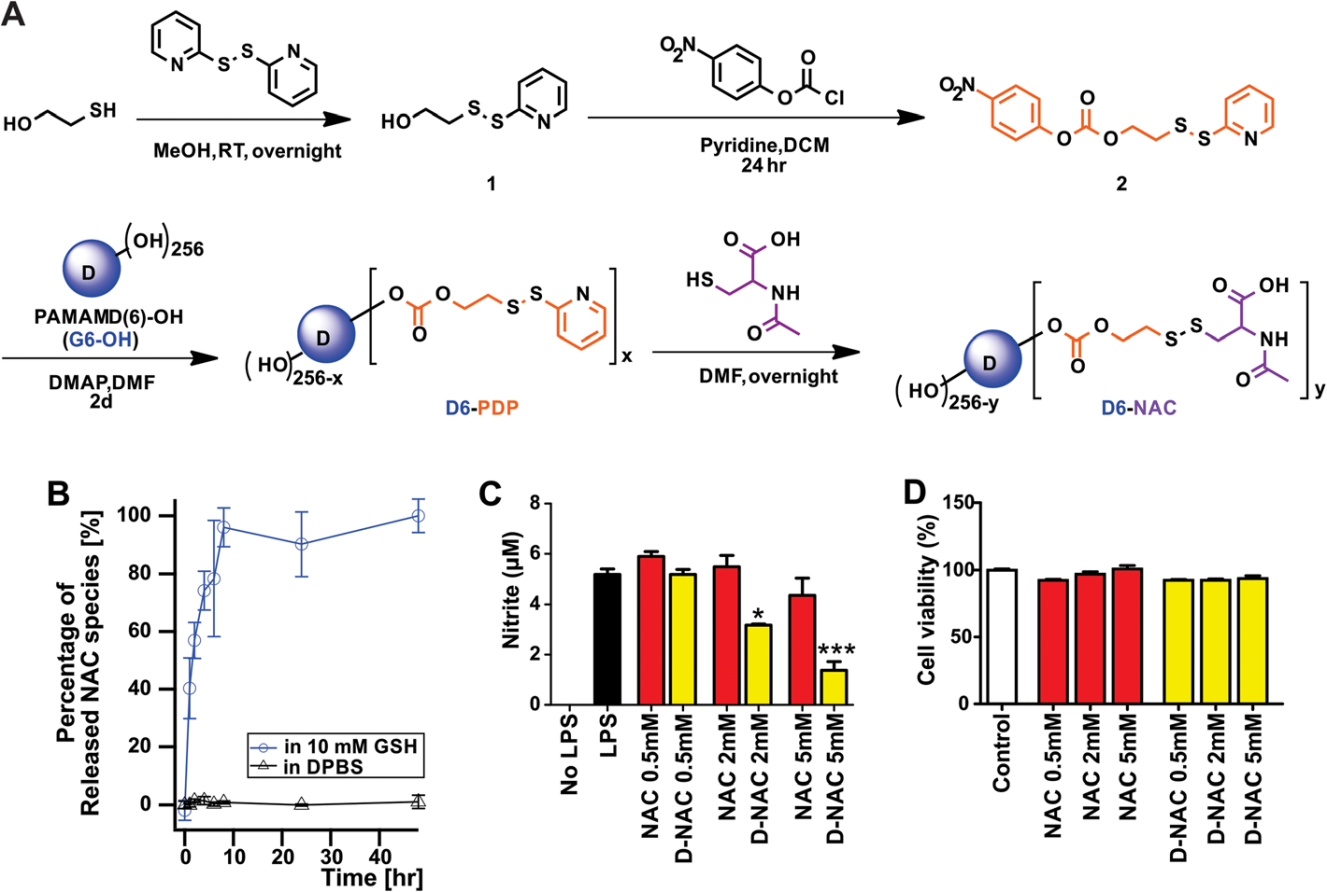
Synthesis and in vitro characterization of G6D-NAC. **a** Synthesis scheme for G6D-NAC. **b** Release profile of G6D-NAC when incubated with 10 mM GSH or in DPBS at 37 °C. ~ 90% of the NA C is released from G6D-NAC in 8 h at this level of GSH (*n* = 3). **c** G6D-NAC is more effective in decreasing NO production from LPS exposed BV2 cells in vitro when compared to NAC alone. **d** Cytotoxicity assessment of G6D-NAC and free NAC at 0.5–5 mM (based on NAC concentrations). BV-2 cells were pre-activated by LPS for 3 h and incubated with dendrimer or free drug for an additional 24 h. Cell viability was determined by MTS cell proliferation assay kit and presented as the viable cell percentage normalized to the control cells (without dendrimer/dendrimer conjugate treatment). *p < 0.05, ***p < 0.001

The synthesis of G6D-NAC was accomplished first by conjugating a carbonate linker, **2**, with hydroxyl groups on G6 dendrimers under the catalysis of dimethylaminopyridine (DMAP) to form a carbonate ester bond between the linker and the dendrimer, garner pyridyldisulfanyl groups, and form an intermediate G6D-PDP; this intermediate underwent disulfide exchange with NAC to give the final product G6D-NAC (Fig. 1a). The synthesis of the carbonate linker **2** was based on a reported work [31, 38]. This synthetic protocol could allow approximately ~ 125 NAC molecules to be conjugated to one G6 dendrimer molecule according to NMR spectra (the drug loading was calculated to be ~ 23% w/w). The hydrodynamic size of G6D-NAC was 8.5 nm according to dynamic light scattering (DLS). The overall purity (99%) of the materials was assessed by analytical reverse phase high performance liquid chromatography (RP-HPLC), with G6D-NAC eluting at 15.2 min [42].

### NAC is released from G6D-NAC by intracellular levels of glutathione

For this conjugate, the GSH-triggered release of NAC was investigated by incubating G6D-NAC with 10 mM GSH (intracellular GSH levels) at 37 °C, and then analyzing the released products with reverse phase HPLC. The main products formed after treating with GSH composed of three main NAC-associated products: NAC, GSH-NAC complex, and NAC-NAC dimer [43]. The NAC release percentage was calculated by normalizing the sum of the area under the curve (AUC) on chromatograms of all NAC-associated species (NAC, GSH-NAC, and NAC-NAC) by the AUC when equilibrium was reached after 48 h. About 74% of conjugated NAC was released in these three forms in the first 4 h upon GSH treatment, while over 90% was released after 8 h in 10 mM of GSH (Fig. 1b). Without the treatment of GSH, these species were released less than 1% in DPBS in 48 h. In the presence of excess amounts of GSH, GSH-NAC (75%) was the major species formed after NAC was released from the dendrimer, followed by NAC-NAC (15%) and NAC monomer (10%).

### G6D-NAC is more effective in decreasing oxidative stress and is not cytotoxic

The anti-oxidative effect of G6D-NAC was investigated in an in vitro LPS-activated murine microglial BV-2 cell line, by measuring nitrite (NO) levels. BV-2 cells were pre-activated by 100 ng of LPS for 3 h followed by G6D-NAC or NAC treatment (0.5, 2, or 5mM) for an additional 3 h. NO levels were measured using a Nitrate/Nitrite Colorimetric Assay Kit. A dose response was noted with a decrease in nitrite levels with increasing doses of G6D-NAC. G6D-NAC was more effective in decreasing nitrite levels at 2 and 5 mM and was significantly better than equivalent dose of the free NAC (Fig. 1c). This suggests that dendrimer delivers the drug better into the target cells [44].

We evaluated the cytotoxicity of G6D-NAC at the corresponding NAC concentrations (0.5–5.0 mM) by incubating NAC or G6DNAC for 24 h with BV-2 cells (pre-activated with LPS for 3 h, without follow-up LPS removal). MTS [3-(4,5-dimethylthiazol-2-yl)-5-(3-carboxymethoxyphenyl)-2-(4-sulfophenyl)-2H-tetrazolium ] cell proliferation assay showed that there was no significant difference in cell viability at the different concentrations (*p* > 0.05), suggesting that G6D-NAC was not cytotoxic at this concentration range (Fig. 1d).

### G6D-Cy5 localizes with activated microglia in CP kits

**Fig. 2:**
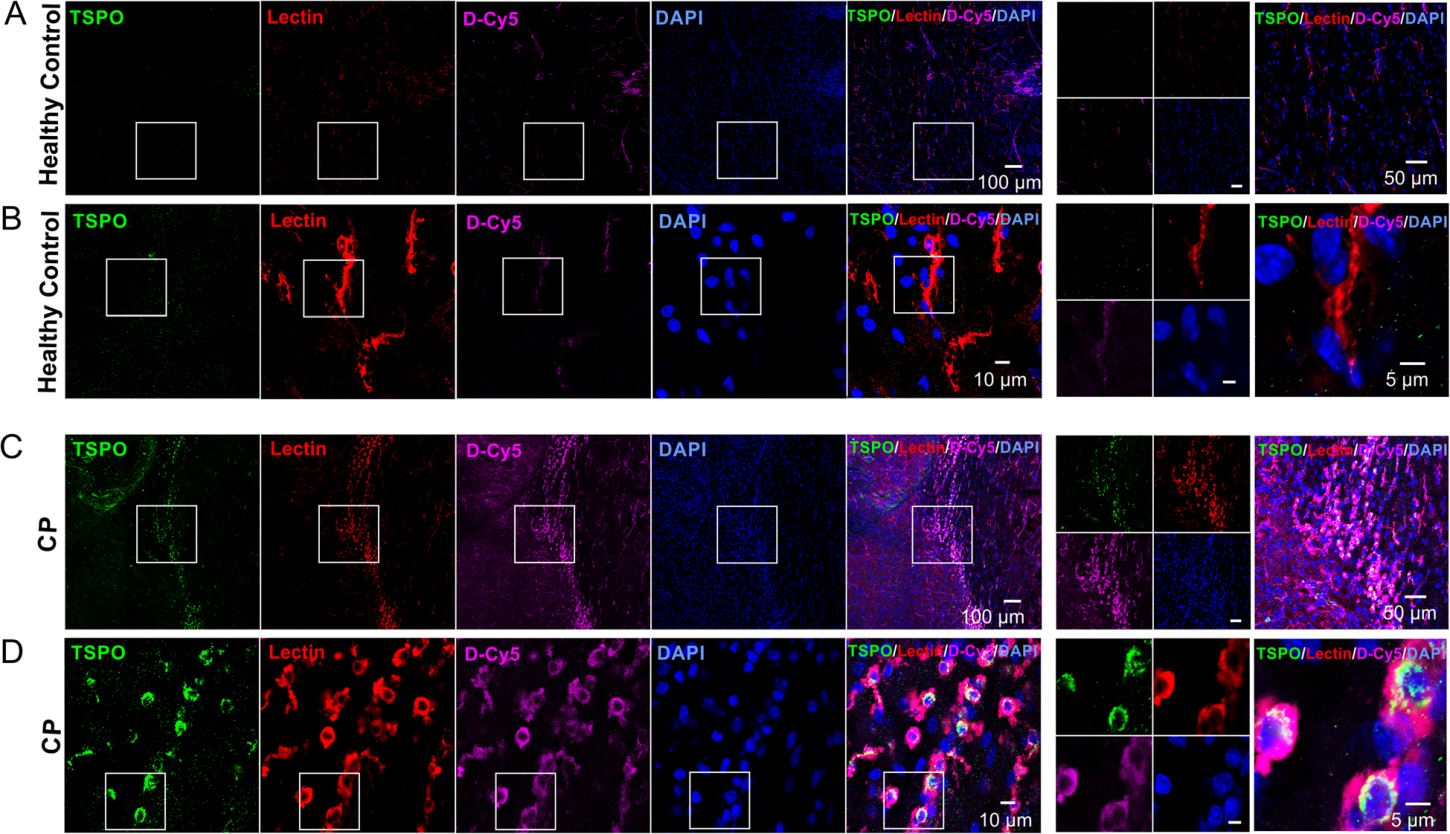
G6D-Cy5 and microglial co-localization in healthy controls and CP kits. Brain slices were stained with TSPO and lectin (microglial marker). Images were acquired from the corpus callosum (white matter) of healthy control and CP kits. Very weak G6D-Cy5 signal was detected in the microglia in healthy control kits (**a**, **b**). Intense G6D-Cy5 signal is mainly co-localized with activated microglia at the corpus callosum in CP kits (**c**, **d**). Right panels are the higher magnification images indicated by the boxes on the left panes of images. Moreover, TSPO expression is higher in activated microglia in CP kits

Healthy control (*n* = 3) and CP kits (*n* = 3) received an intravenous administration of G6D-Cy5 on PND1 and were sacrificed 24 h post-injection. We found that the Cy5-labeled dendrimer-drug conjugates co-localized with activated microglia, indicated by ameboid soma with shortened processes and increased expression of TSPO, in the periventricular white matter region (PVR) in the cortex and the white matter areas in cerebellum of CP kits. An example of localization of Cy5 with activated microglia in the corpus callosum is shown in Fig. 2.

### A single dose of systemically administration G6D-NAC improves motor function in CP kits at PND5

**Fig. 3:**
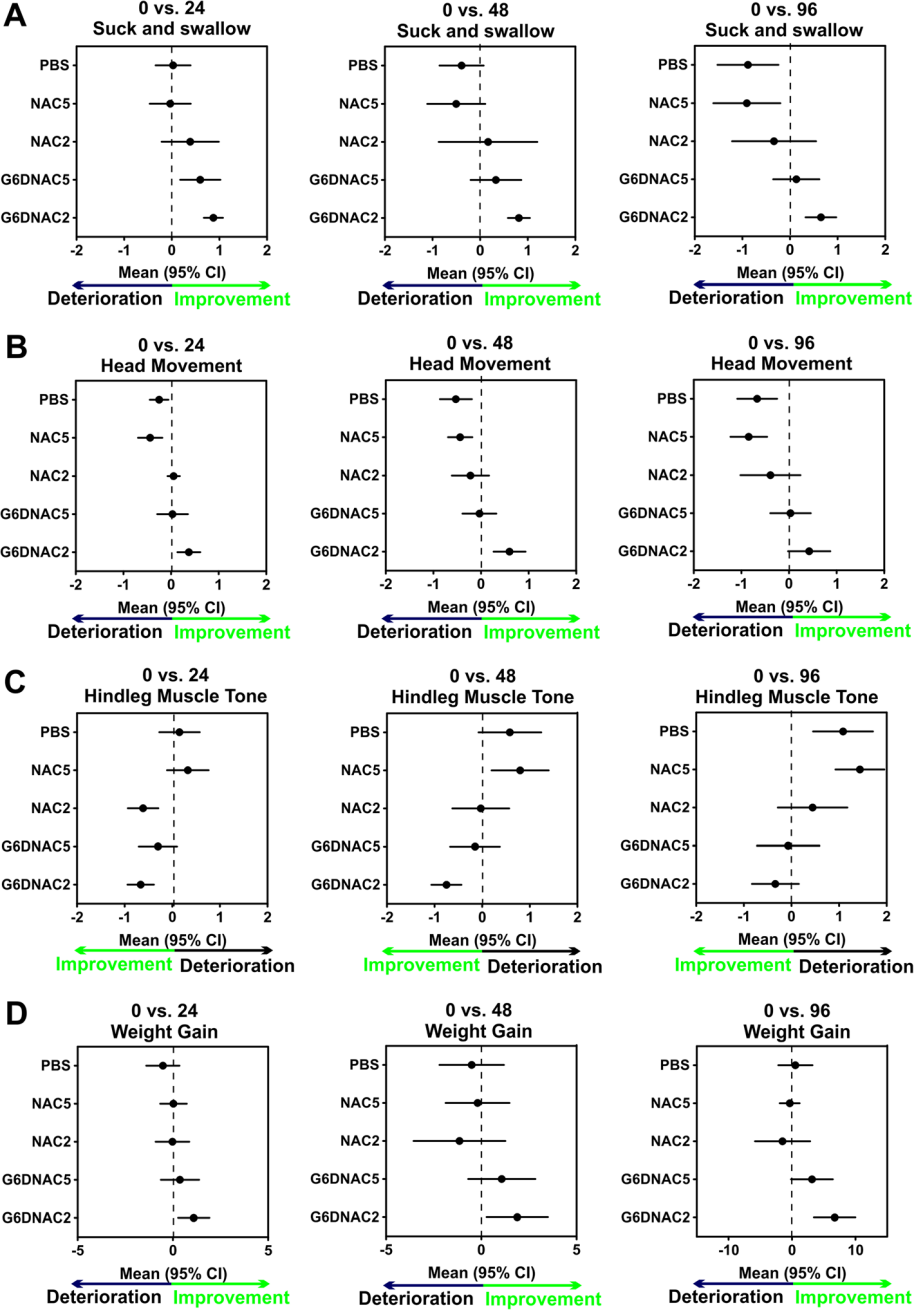
Changes in motor function and weight gain post-treatment in CP kits. The CP kits were randomly divided into 5 groups and received systematic injections of PBS, NAC (2, 5 mg/kg) or G6D-NAC (2, 5 mg/kg). The behavioral scores were assessed at 24, 48, and 96 h post-injection. The suck and swallow (**a**), head movement (**b**), and hindleg muscle tone (**c**) were significantly improved in G6D-NAC2 group at 48 and 96 h post-treatment. Moreover, G6D-NAC 2 mg/kg treatment significantly increased weight gain (**d**) at 96 h post-treatment. **p < 0.01, ***p < 0.001, G6D-NAC2 vs. PBS, NAC2, and NAC5 groups

CP littermates were randomly divided in to 5 groups: PBS (*n* = 18, from 12 litters), NAC2 (*n* = 9, from 8 litters), G6D-NAC2 (*n* = 20, from 14 litters), NAC5 (*n* = 16, from 11 litters) and G6D-NAC5 (*n* = 15, from 11 litters). On postnatal day 1 (PND1, 3 days post-injury) the kits were intravenously administrated PBS, NAC 2 mg/kg, G6D-NAC 2 mg/kg, NAC 5 mg/kg, or G6D-NAC 5 mg/kg (calculated based on the corresponding NAC contents), respectively. These doses were selected based on our previously published biodistribution data [36]. We found that accumulation of G6OH in the brain was at least twice that of G4OH [31]. Healthy controls (*n* = 15, from 3 litters) did not receive any treatment and served as naïve controls. Motor functions were evaluated at 24, 48, and 96 h post-injection by personnel blinded to the experiments. Our results indicate that the baseline motor function of CP kits was similar among CP groups at PND1, which represented a mild to moderate phenotype as previously defined by our group (Supplemental Table 1) [16, 32]. Upon two-way analysis of variance (time × treatment), we found that a single dose of G6D-NAC 2.0 mg/kg significantly improved motor function, including increased scores of suck/swallow [time: *F*_(1,219)_ = 5.52, *p* = 0.0003; treatment: *F*_(1,219)_ = 22.95, *p* < 0.0001; time × treatment: *F*_(1,219)_ = 1.14, *p* = 0.89] and head movements [time: *F*_(1,219)_ = 1.58, *p* = 0.09; treatment: *F*_(1,219)_ = 26.01, *p* < 0.0001; time × treatment: *F*_(1,219)_ = 2.14, *p* = 0.57], as well as decreased hindlimb muscle tone [time: *F*_(1,219)_ = 6.5, *p* < 0.0001; treatment: *F*_(1,219)_ = 24.4, *p* < 0.0001; time × treatment: *F*_(1,219)_ = 2.03, *p* = 0.58] at 48 and 96 h post-injection (Fig. 3a–c). We also monitored the body weight of all kits and found that baseline body weight was similar among all CP groups at PND1 (*p* > 0.05) (Supplemental Table 1). G6D-NAC 2.0 mg/kg significantly increased weight gain at PND5 (7 days post-injury and 96 h post-treatment) compared with PBS, NAC2, and NAC5 groups. The body weight in the G6D-NAC5 group also significantly increased, compared with NAC2 and NAC5 groups [time: *F*_(1,219)_ = 2.58, *p* = 0.03; treatment: *F*_(1,219)_ = 12.78, *p* < 0.0001; time × treatment: *F*_(1,219)_ = 6.33, *p* = 0.02] (Fig. 3d). No significant differences were noted between the G6D-NAC2 and the G6D-NAC5 groups.

### Single dose of G6D-NAC decreased microglial activation in both PVR and cerebellum of CP kits at PND5

**Fig. 4:**
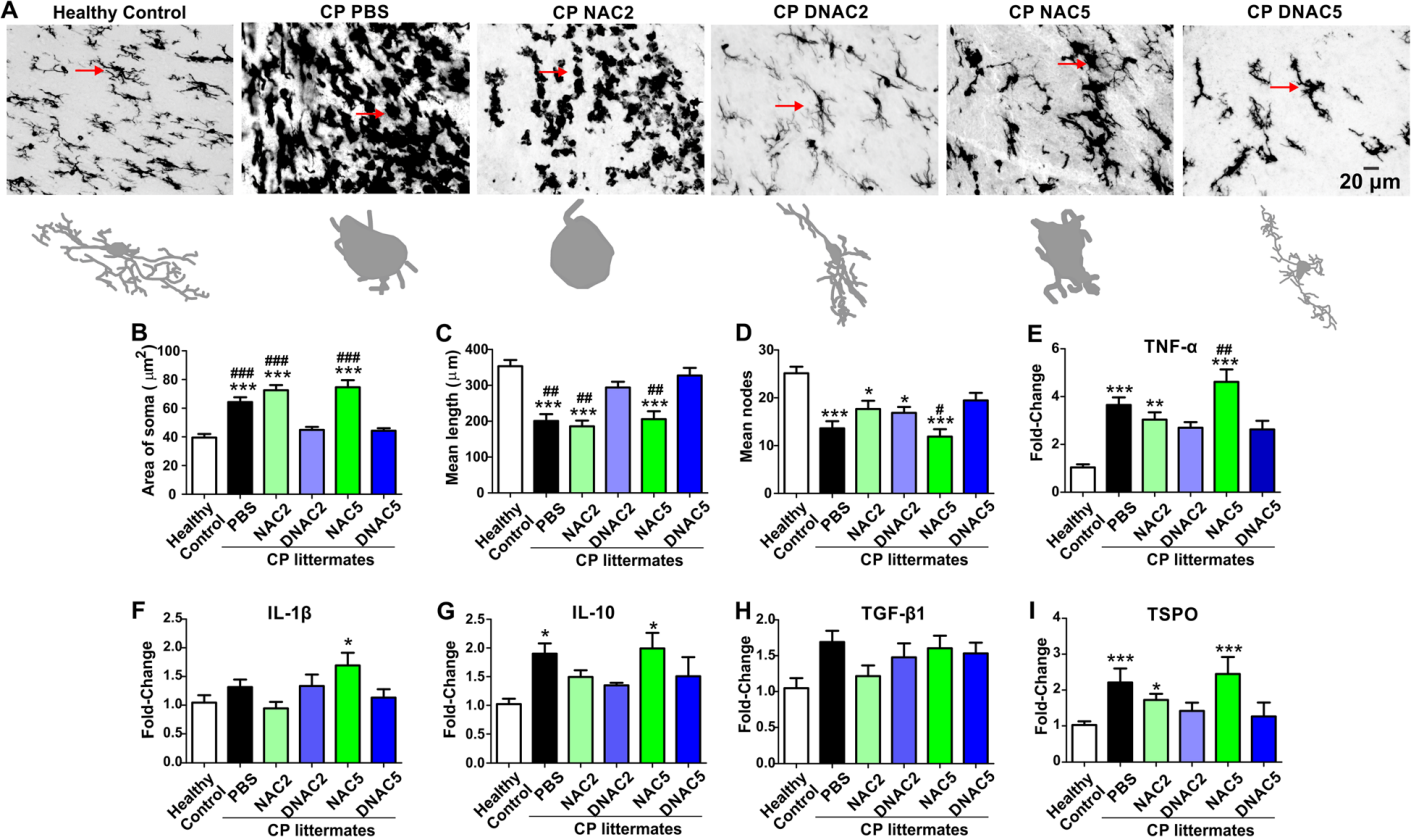
Microglial morphology and mRNA expressions of cytokines and TSPO in the PVR at PND5. **a** Brain slices were stained with IBA1 antibody (microglial marker), and images (× 40) were randomly acquired from the corpus callosum from healthy controls, PBS, NAC2, G6D-NAC2, NAC5, and G6D-NAC5 groups and analyzed using the Neurolucida and Neurolucida Explorer software. The arrows indicate the traced cells. Scale bar, 20 μm. The G6D-NAC (2 and 5 mg/kg) treatment significantly decreased the soma size (**b**), the length (**c**), and the nodes (**d**) of the processes of the microglia, in comparison with PBS and NAC (2 and 5) groups. In addition, G6D-NAC (2 and 5 mg/kg) treatment decreased TNF-α (**e**) and IL-10 (**g**) mRNA expressions. No significant changes in the mRNA expression of IL-1β (**f**), TGF-β1 (**h**), and TSPO (*I*). *p < 0.05, **p < 0.01, ***p < 0.001, compared with healthy controls. #*p* < 0.05, ##*p* < 0.01, ###*p* < 0.001, compared with G6D-NAC2 and G6D-NAC5

We measured the microglia morphology in the PVR (periventricular region) from healthy control (*n* = 7, from 3 litters), PBS (*n* = 7, from 6 litters), NAC2 (*n* = 6, from 5 litters), G6D-NAC2 (*n* = 6, from 6 litters), NAC5 (*n* = 7, from 5 litters), and G6D-NAC5 (*n* = 5, from 3 litters) kits. Upon one way-ANOVA analysis, we found a significant increase in the soma size and a decrease in the length and nodes of microglial processes in the PBS animals when compared to healthy controls, with no improvement noted in the free NAC2 and free NAC5 groups. G6D-NAC (2.0 and 5 mg/kg) treatment significantly decreased the soma size [*F*_(1,239)_ = 22.08, *p* < 0.0001] and significantly increased the length of the processes [*F*_(1,239)_ = 13.94, *p* < 0.0001] and was similar to the healthy control level (Fig. 4a–c). G6D-NAC 5.0 mg/kg, significantly increased the nodes of processes [*F*_(1,239)_ = 7.45, *p* < 0.0001], similar to the healthy controls (Fig. 4d).

We also measured the mRNA expressions of cytokines and TSPO (a maker of microglial activation). We found that mRNA expressions of TNF-α (tumor necrosis factor alpha) [*F*_(1,39)_ = 11.5, *p* < 0.0001] and TSPO [*F*_(1,39)_ = 2.61, *p* = 0.04] significantly increased in the PBS kits when compared to healthy controls. Treatment with NAC 2.0 mg/kg and NAC 5.0 mg/kg showed no improvement. G6D-NAC (2.0 and 5.0 mg/kg) treatment significantly decreased TNF-α mRNA expression, when compared with PBS and NAC2 and NAC5 groups (Fig. 4e). G6D-NAC (2.0 and 5.0 mg/kg) treatment showed a trend of decreased TSPO mRNA expression, but did not reach statistical significance (Fig. 4i). There was no significant difference in mRNA expression of IL-1β (interleukin-1 beta), IL-10 and TGF-β1 (transforming growth factor beta 1) between the groups by PND 5 (Fig. 4f–h).

**Fig. 5:**
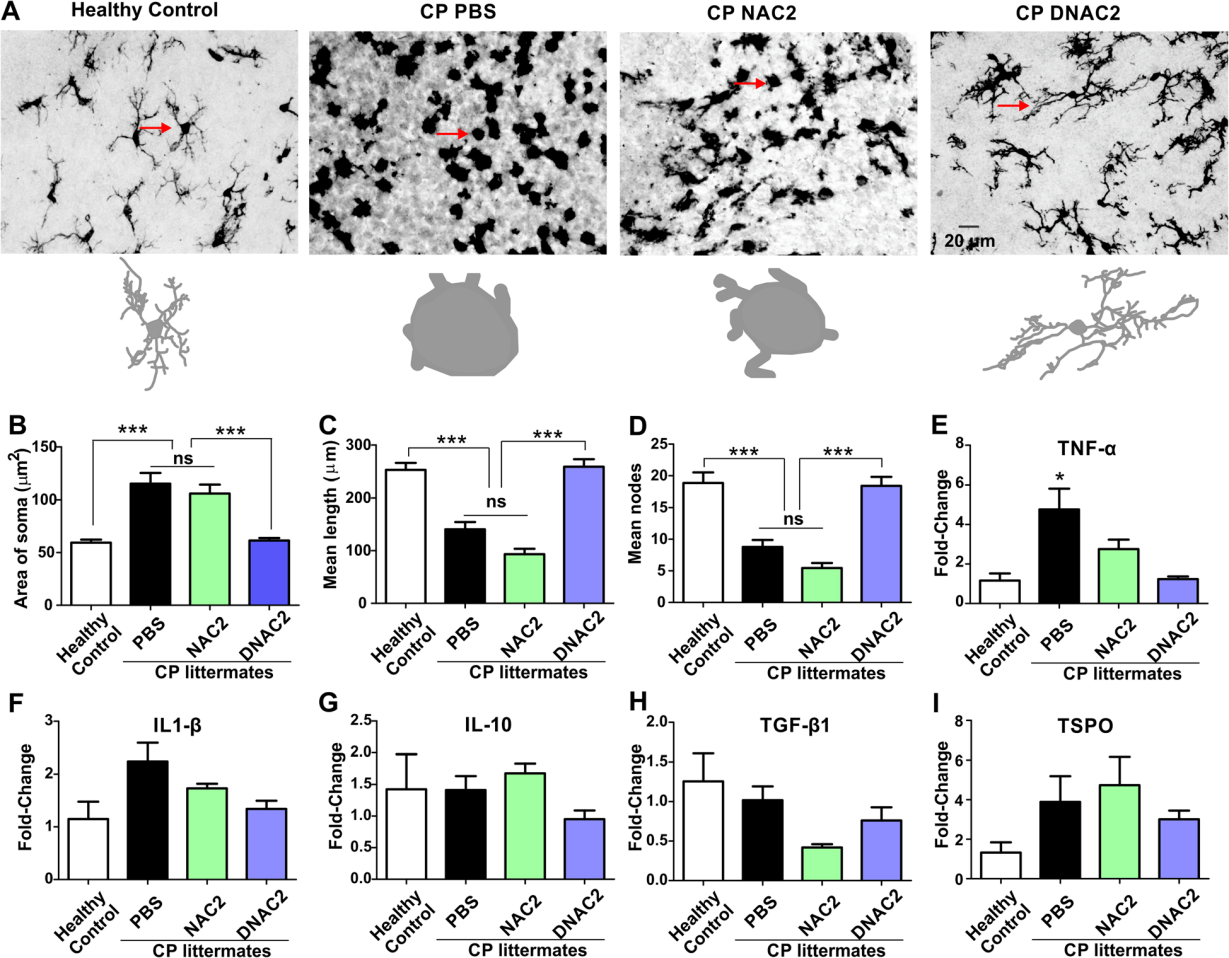
Dendrimer-NAC treatment ameliorated the microglial activation in the cerebellar white matter areas of CP kits on PND5. Brain slices were stained with IBA1 antibody (microglial marker) and images (× 40) were randomly acquired from the white matter areas from healthy controls and endotoxin kits treated with PBS, NAC (2 mg/kg), and G6D-NAC (2 mg/kg). **a** Representative microglia images. The arrows indicate the microglia that correspond to the Neurolucida tracing images. **b**–**d** Microglial morphological analysis. The microglia in the endotoxin PBS and NAC2 groups had significantly increased area of the soma (**b**) and significant decreased mean length (**c**) and mean node (**d**) of the processes, compared to the healthy control kits. The microglial morphology, including the area of soma and the length and nodes of processes in the D-NAC2 group, were similar to those of healthy controls. **e** The TNF-α mRNA expression significantly increased in the endotoxin PBS group. Compared with that in the NAC2 group, the TNF-α mRNA expression significantly decreased in the D-NAC2 group and reached a similar level as in that of healthy controls. There was no significant change in mRNA expression of IL-1β (**f**), IL-10 (**g**), TGF-β1 (**h**), and TSPO (**i**). *p < 0.05, ***p < 0.001, compared with healthy controls. ns no significance

We further compared the microglial morphology in the cerebellum in healthy control (*n* = 4, from 3 litters), CP PBS (*n* = 6, from 5 litters), CP NAC2 (*n* = 3, from 3 litters), and CP D-NAC2 (*n* = 6, from 6 litters) at PND5. Since D-NAC 2.0 mg/kg and D-NAC 5.0 mg/kg had similar effects in normalizing microglial morphology in the cortex, we analyzed just the D-NAC 2.0 mg/kg and NAC 2.0 mg/kg treatment groups. Upon one way-ANOVA analysis, we found that the size of the soma was significantly increased [*F*_(1,159)_ = 18.86, *p* < 0.0001] (Fig. 5a, b) and the nodes [*F*_(1,159)_ = 26.34, *p* < 0.0001] and length [*F*_(1,159)_ = 34.74, *p* < 0.0001] of the microglial processes were significantly decreased in the CP PBS groups when compared to control kits without any improvement noted with the free drug treatment (Fig. 5c, d). On the other hand, the soma size, the nodes, and the length of the processes in the D-NAC2 group were significantly better than PBS and free NAC treatment and was similar to the healthy control group (Fig. 5a–d). mRNA expressions of cytokines and TSPO demonstrated a significant increase in TNF-α in the CP PBS group (*n* = 10, from 8 litters) compared with healthy controls (*n* = 5, from 5 litters). A significant decrease in the TNF-α mRNA expression was seen in D-NAC2 group (*n* = 5, from 4 litters) when compared to PBS treated animals and was similar to that of the healthy control group [*F*_(1,26)_ = 3.88, *p* = 0.02], while free NAC2 (*n* = 6, from 5 litters) had no effect (Fig. 5e). However, there was no significant differences in the mRNA expression of IL-1β, IL-10, TGF-β1, and TSPO (*p* > 0.05) (Fig. 5f–i).

### G6D-NAC treatment improved survival rate of CP kits at PND15

**Fig. 6:**
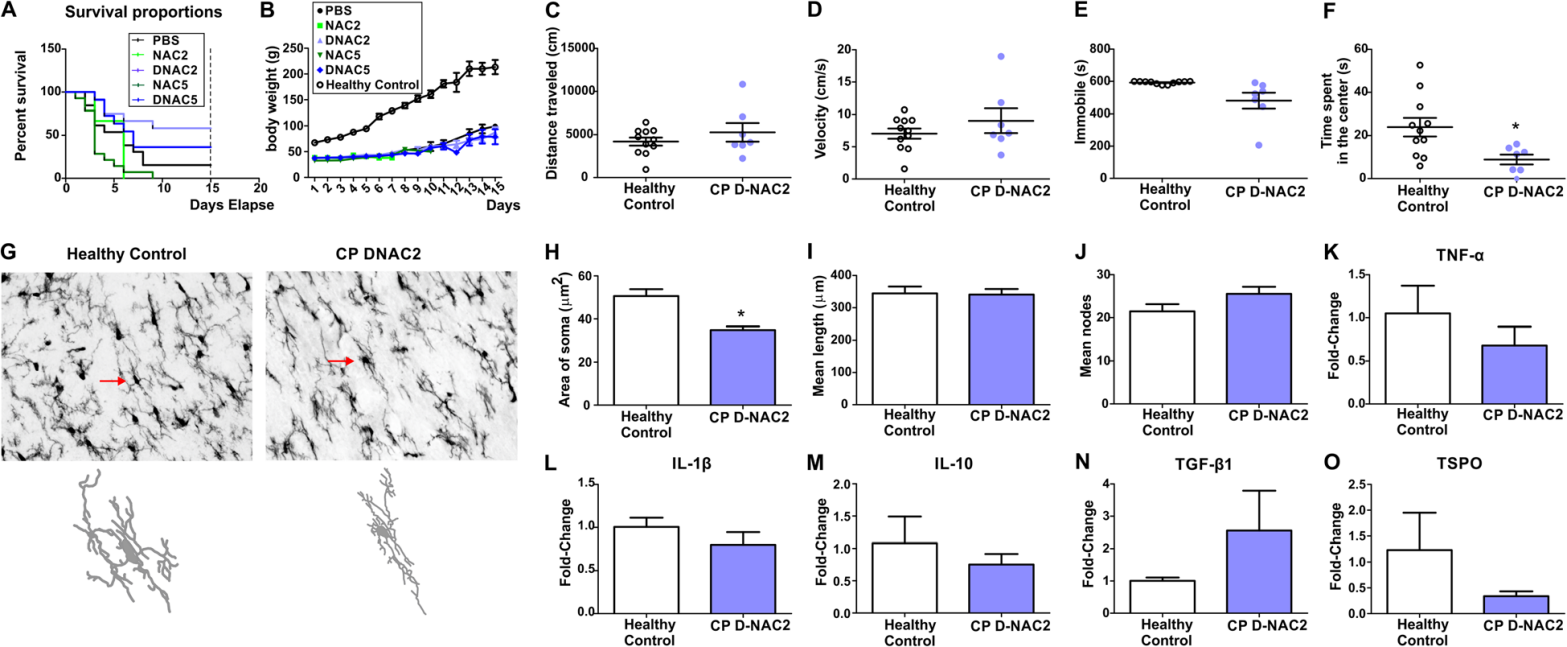
The efficacy of G6D-NAC treatment at PND15. **a** Survival rate at PND15 from all groups. **b** Body weight of healthy control, PBS, NAC2, NAC5, G6D-NAC2, and G6D-NAC5 groups. **c**–**f** Open field test on motor function and anxiety in healthy controls and G6D-NAC2. There was no significant difference in the distance traveled (**c**) and velocity (**d**) among these groups. However, the immobile time (**e**) and the time spent in the center (**f**) significantly decreased in the G6D-NAC2 group. *p < 0.05, compared with healthy controls. **g** Microglial morphology at PND15. Brain slices were stained with IBA1 antibody (microglial marker) and images (× 40) were randomly acquired from the corpus callosum from healthy controls and G6D-NAC2 groups and analyzed using the Neurolucida and Neurolucida Explorer software. The arrows indicated the traced cells. Scale bar, 20 μm. The area of the soma significantly decreased in the G6D-NAC2 group (**h**). There was no difference in the mean length (**i**) and the mean nodes (**j**) of the processes of the microglia among the groups. In addition, there were no differences in the mRNA expression of TNF-α (**k**), IL-1β (**l**), IL-10 (**m**), TGF-β1 (**n**), and TSPO (**o**). *p < 0.05, compared with healthy controls

We compared the survival rate of the CP kits at PND15, which was 18.2% (2/11, from 9 litters) in the PBS group, 0% (0/6, from 6 litters) in NAC2, 0% (0/12, from 11 litters) in NAC5, 58.3% (7/12, from 9 litters) in G6D-NAC2, and 33.3% (4/12, from 10 litters) in G6D-NAC5, respectively (Fig. 6a).

We also compared the daily body weight (PND1-15) of all CP groups with the healthy controls (*n* = 11, from 6 litters). We found that kits in all CP groups had significantly lower body weight at PND1, compared with the healthy controls (Fig. 6b, Table 2). Upon the analysis of two-way ANOVA, the body weight of kits in all CP groups was significantly less than the that of healthy controls over time (from PND1 to PND15) [time: *F*_(1,219)_ = 2.58, *p* = 0.03; treatment: *F*_(1,219)_ = 12.78, *p* < 0.0001; time × treatments: *F*_(1,219)_ = 6.33, *p* = 0.02], and there was no significant difference among CP groups (Fig. 6b). In addition, we monitored the maturation levels of kits from healthy control and G6D-NAC2 groups (other CP groups were not included due to their low survival rate). The G6D-NAC2 group had significant survival to enable this analysis, but the kits had significantly delayed milestones, such as eye opening, head and body elevation, and normal gait (Table 2), compared to healthy controls.

**Table 2:** Maturation onset (days) in healthy control and G6D-NAC2 groups

Developmental milestone	Healthy control (*n* = 11)	G6D-NAC2 (*n* = 7)
Eye opening	10 (9, 10)***	13 (13, 14)
Body elevation	8 (7, 8)***	12 (11, 14)
Normal gait	8 (7, 8)***	12 (11, 13)
Head elevation	8 (7, 8)***	13 (11, 14)
Cessation of falling	7 (6, 7)***	13 (12, 15)

Data were expressed as median (inter-quartile range). Body elevation, simultaneous fore- and hind-limb elevation; head elevation, head elevated for more than 1 min; cessation of falling, locomotion without falling. *p* values for other milestones were analyzed by one-way ANOVA (non-parametric)

***p < 0.0001, compared with G6D-NAC2 and G6D-NAC5

### CP kits in G6D-NAC group show similar motor function and microglial activation as healthy controls at PND15

Because of the low survival rate of kits from PBS (*n* = 2 out of 11), free NAC2 (*n* = 0 out of 9), free NAC5 (*n* = 0 out of 12), and G6D-NAC5 (*n* = 4 out of 12) groups at PND15, we only compared the motor function of the kits in healthy controls (*n* = 11, from 6 litters) and G6D-NAC2 treated CP animals (*n* = 7, from 6 litters) groups using an open field test. We found that the distance traveled, velocity, and immobile time were similar (*p* > 0.05) (Fig. 6c–e); however, the time spent in the center of the arena significantly decreased in the G6D-NAC 2 group (*p* = 0.0008) (Fig. 6f).

We also measured the microglial morphology in healthy controls (*n* = 5, from 3 litters) and G6D-NAC2 (*n* = 6, from 5 litters). We found that there was a significant decrease in the soma size in D-NAC2 group (*p* < 0.0001), but there was no difference in length and nodes of processes of microglia (Fig. 6g–j). Moreover, there were no significant differences in the mRNA expression of TNF-α, IL-1β, IL-10, TGF-β1, and TSPO between G6D-NAC2 and healthy control (Fig. 6k–o).

## Discussion

In this study, we successfully conjugated and characterized the in vitro efficacy of G6D-NAC conjugate (containing a high payload of ~ 125 NAC molecules per dendrimer). We further evaluated the long-term efficacy of G6D-NAC in a clinically relevant in vivo rabbit CP model. Multiple measures of efficacy following a single intravenous dose of G6D-NAC on PND1 in the treatment of CP kits were assessed, for the very first time, over a 15-day period. In this study, CP kits treated with free NAC died before PND9, whereas G6D-NAC-treated CP kits survived beyond PND15.

In LPS-activated BV-2 microglial cells, G6D-NAC was significantly better than free NAC in addressing oxidative stress, suggesting that the dendrimer transports NAC into cells, delivering superior drug efficacy [24]. We also showed that the conjugation of a large number of NAC molecules to the G6 dendrimer (125 NAC molecules out of 256 surface hydroxyl groups) did not alter the ability of the conjugate to target the activated microglial cells in vivo (Fig. 2). The biodistribution in the brain was qualitatively similar to vehicle hydroxyl G4 and G6 dendrimers [31]. In this study, the CP kits had a mild/moderate phenotype, which is different from the severe CP phenotype that was used in our previous studies [16, 19, 20, 32]. The advantage of using mild/moderate CP phenotype is that the CP kits are able to survive longer than the severe CP phenotype, allowing us to monitor survival, neurobehavioral changes, and deficient maturations during development. Interestingly, although the CP kits had mild to moderate phenotype at PND1, their motor function deteriorated over time and most of the CP kits in the PBS and free NAC (2.0 and 5.0 my/kg) groups died before PND9, which might be due to continued neuroinflammation, increased glutamate excitotoxicity, impaired myelination, and neuronal loss [16, 19, 39].

In contrast to the free NAC, systematic administration of a single dose of G6D-NAC (containing the same amount of 2.0 and 5 mg/kg NAC) on PND1 (the day of birth, 3 days post-initial injury) significantly ameliorated pro-inflammatory microglial activation, indicated by decreased soma size and increased length and nodes of the processes of the microglia, and decrease mRNA expressions of pro-inflammatory cytokine TNF-α and TSPO (upregulated in activated microglia) in the periventricular and cerebellar white matter areas. Moreover, 2 mg/kg D-NAC treatment significantly improved motor function, such as suck/swallow, head movement, and hindlimb muscle tone at PND5 (7 days post-injury), increased survival rate, and achieved similar motor function, patterns of microglial activity, and pro-inflammatory cytokine expression as healthy controls at PND15 (17 days post-injury), in comparison with PBS and free NAC treated groups. Interestingly, the CP G6D-NAC2 kits spend less time in the center of the maze, compared to healthy controls. This might be due to the delayed maturation, such as in eye opening. This is the first time that the effects of D-NAC therapy are analyzed over 15 days in our CP rabbit model. A single dose of DNAC administered systemically in the postnatal period appears to induce significant sustained therapeutic effects. Therefore, D-NAC conjugates provide an effective therapeutic platform for neonatal brain injuries.

The superior efficacy of the G6D-NAC conjugate might be due to its high drug payload (~ 125 NAC/dendrimer) and longer circulation time [36]. We have previously shown that the longer circulation time of the G6OH dendrimer leads to greater accumulation in Iba1-stained ‘activated’ microglia/macrophages in various models of neuroinflammation [31, 36, 37]. NAC exhibits neuroprotective effects in animal models of chorioamnionitis by decreasing oxidative stress and pro-inflammatory cytokine production, increasing intracellular glutathione level, and preventing hypomyelination [45–49]. However, in a fetal sheep model of septic shock, antenatal NAC administration increased fetal hypoxemia [50]. In human infants and pregnant mothers exposed to chorioamnionitis, antenatal and postnatal NAC showed anti-inflammatory and neuroprotective effects without severe side effects [51]; however, NAC has a low oral bioavailability requiring high doses and frequent re-dosing [51, 52]. When administered intravenously, NAC binds to plasma proteins via covalent disulfide bonds and can also cause allergic reactions in some patients complicating its use [53]. Moreover, since G6D-NAC would bypass the cystine-glutamate antiporter, the potential increase in extracellular glutamate and associated excitotoxicity seen with free NAC would be avoided [24]. The D-NAC conjugate is transported across the blood brain barrier, and delivers the NAC to activated microglia in a cell-specific manner, thereby increasing NAC efficacy and decreasing unwanted side effects. Compared with 2 mg/kg D-NAC treatment, 5 mg/kg D-NAC treatment did not show a better efficacy. It might indicate that 2 mg/kg D-NAC already achieved the maximum treatment efficacy. However, it is possible that higher dosage of free NAC can result in detrimental side effects as seen in the fetal sheep model of septic shock [50], although we would not expect that with the dendrimer-conjugated NAC. It is also possible that there are sex-related differences in the response to therapy, which we have not interrogated here. This will be evaluated in future studies.

Although a single dose of G6D-NAC treatment significantly improved functionality, it did not improve the CP kits’ body weight and delayed maturation. On the one hand, it might indicate a need for re-dosing to further decrease neuroinflammation. Moreover, other factors such as glutamate excitotoxicity and dysregulation of serotonin level [16, 20] that play important roles in the development of immature brain may be negatively impacting these outcome measures. It might also be necessary to use combination therapies to target multiple pathways in the CP pathophysiology to improve other aspects of development.

## Conclusion

Our study indicates that a single, low dose of G6D-NAC significantly decreases neuroinflammation and improves motor function in the developing brain over a sustained period, providing a promising platform for nanoparticle-guided drug delivery in the treatment of pediatric brain injuries.

## Supplementary information

**Additional file 1: Supplemental Table 1.** Baseline (pretreatment) neurobehavior test scores and body weight at PND1

## Data Availability

The datasets used and/or analyzed during the current study are available from the corresponding author on reasonable request.

## Abbreviations

BBB: Blood-brain-barrier
CNS: Central nervous system
CP: Cerebral palsy
G6: Generation-6
G6D-NAC: G6-PAMAM dendrimer-NAC
G6-OH: Generation 6 hydroxyl-terminated PAMAM dendrimer
GAPDH: Glyceraldehyde 3-phosphate dehydrogenase
GSH: Glutathione
IBA-1: Ionized calcium-binding adaptor molecule 1
IL: Interleukin
LPS: Lipopolysaccharide
MTS: 3-(4,5-Dimethylthiazol-2-yl)-5-(3-carboxymethoxyphenyl)-2-(4-sulfophenyl)-2H-tetrazolium
NAC: *N*-acetyl-l-cysteine
NO: Nitrite
PAMAM: Poly(amidoamine)
PBS: Phosphate-buffered saline
PDA: Photodiode array
PND: Postnatal day
PVR: Periventricular region
TGF-β1: Transforming growth factor beta 1
TNF-α: Tumor necrosis factor alpha
TSPO: Translocator protein
