# Supplementary material for: Systemic dendrimer-drug nanomedicines for long-term treatment of mild-moderate cerebral palsy in a rabbit model
Source: J Neuroinflammation. 2020 Oct 25;17:319. doi: 10.1186/s12974-020-01984-1 (PMC7586697; doi:10.1186/s12974-020-01984-1)
Supplement: Supplementary file 1 — Additional file 1: Supplemental Table 1. Baseline (pretreatment) neurobehavior test scores and body weight at PND1 [file 12974_2020_1984_MOESM1_ESM.docx]

**Supplementary information:**

**Supplemental Table 1. Baseline (pretreatment) neurobehavior test scores and body weight at PND1.**

|  | **Groups** | | | | | |
| --- | --- | --- | --- | --- | --- | --- |
| **Outcomes** | **Healthy Control**  **(n=15)** | **Endotoxin groups** | | | | |
|  |  | **PBS**  **(n=18)** | **NAC2**  **(n=9)** | **G6D-NAC2**  **(n=20)** | **NAC5**  **(n=16)** | **G6D-NAC5**  **(n=15)** |
| Posture | 3.0 (3.0, 3.0)*** | 2.0 (2.0, 2.5) | 2 (2.0, 2.5) | 2.0 (2.0, 2.5) | 2.0 (2.0, 2.0) | 2.0 (2.0, 2.0) |
| Suck and Swallow | 3.0 (3.0, 3.0)*** | 2.3 (2.0, 2.7) | 1.5 (1.5, 2.3) | 2.0 (1.5, 2.0) | 2.3 (1.5, 3.0) | 2.0 (1.5, 2.5) |
| Head movement | 3.0 (3.0, 3.0)*** | 2.0 (1.9, 2.0) | 2.0 (1.5, 2.0) | 1.5 (1.5, 2.0) | 2.0 (1.1, 2.0) | 1.5 (1.5, 2.0) |
| Hindlimb movement | 3.0 (3.0, 3.0)*** | 1.5 (1.0, 2.0) | 2.0 (1.5, 2.0) | 1.5 (1.5, 2.0) | 1.5 (1.5, 2.0) | 1.5 (1.5, 2.0) |
| Hindlimb muscle tone | 0.0 (0.0, 0.0)*** | 1.9 (1.0, 2.1) | 1.5 (1.1, 2.1) | 1.7 (1.1, 2.0) | 1.8 (1.3, 2.0) | 1.8 (1.0, 2.0) |
| Steps | 4.0 (1.0, 4.0)*** | 1.0 (1.0, 1.0) | 1.0 (1.0, 1.0) | 1.0 (1.0, 1.0) | 1.0 (1.0, 1.0) | 1.0 (1.0, 1.0) |
| Aversive response to alcohol | 3.0 (3.0, 3.0)*** | 2.5 (2.0, 3.0) | 2.5 (2.0, 3.0) | 2.5 (2.0, 3.0) | 2.5 (2.0, 3.0) | 2.5 (2.0, 3.0) |
| Body weight (g) | 66.4 (65.1, 71.6)*** | 36.2 (32.6, 40.6) | 37.0 (35.9, 42.1) | 38.0 (34.8, 43.0) | 31.3 (28.9, 37.3) | 37.9 (34.9, 39.4) |

Behavioral scores and body weight of healthy control and endotoxin rabbit kits on PND1. The endotoxin kits had significant impairments in locomotion, olfaction and body weight, compared to healthy control group at PND1. Data were expressed as Median and Percentile. ***p<0.0001, healthy controls *vs.* all endotoxin groups at pre-treatment.
